# Supplementary material for: Bacterial contamination of surgical instruments in 25-gauge pars plana vitrectomy: Incidence and risk factors
Source: PLoS One. 2026 May 28;21(5):e0349663. doi: 10.1371/journal.pone.0349663 (PMC13218455; doi:10.1371/journal.pone.0349663)
Supplement: Attachment — Submitted filename: Response to Reviewers.docx [file pone.0349663.s002.docx]

Dear Editor and Reviewers,

We sincerely thank the Academic Editor and the reviewers for their thorough evaluation of our manuscript and for their valuable and constructive comments. We greatly appreciate the time and effort devoted to reviewing our work.

We have carefully considered all comments and have revised the manuscript accordingly. We believe that these revisions have improved the clarity, robustness, and overall quality of the study. In the following sections, we provide a detailed, point-by-point response to each comment. In the revised manuscript with track changes, deletions are shown using strikethrough, and additions are indicated in red.

The dataset includes clinical information from patients treated at Hiroshima University Hospital. Because these data contain potentially identifiable patient information, public deposition of the dataset is restricted by the Institutional Review Board of Hiroshima University. De-identified data are available from the Hiroshima University Institutional Review Board for researchers who meet the criteria for access to confidential data.

Reviewer #1:

1: Although a 7.3% contamination rate was observed, no cases of postoperative endophthalmitis occurred. A brief discussion clarifying the clinical implications of instrument contamination in the absence of infection would be helpful.

Response:

Thank you for this important comment. We have added a discussion to clarify the clinical implications of bacterial contamination in the absence of postoperative infection. Specifically, we emphasize that bacterial contamination does not necessarily result in clinically apparent endophthalmitis, as a minimum infectious inoculum is required and host defense mechanisms or intraoperative irrigation may eliminate small amounts of bacteria.

(Page10 Lines 247-262)

2: The number of contamination events is small (n = 7). Please comment on the stability of the multivariable analysis and consider acknowledging this limitation more explicitly.

Response:

Thank you for this important comment. We agree that the small number of contamination events may limit the stability of the multivariable analysis. We have revised the manuscript to explicitly acknowledge this limitation and added a statement noting the potential for model overfitting and imprecise estimates due to the low number of events per variable. We have also emphasized that the results should be interpreted with caution and that larger studies are needed to confirm these findings.

Page14 Lines 387-391

3: Four of seven isolates demonstrated fluoroquinolone resistance. While important, the sample size is limited; therefore, conclusions regarding changes in preoperative antibiotic strategy should be presented cautiously.

Response:

Thank you for this important comment. We agree that the number of contamination events is limited and that conclusions regarding changes in preoperative antibiotic strategies should be interpreted with caution. We have revised the Discussion and Conclusion sections to moderate the tone and to emphasize that further studies with larger sample sizes are needed before drawing definitive conclusions regarding antibiotic selection.

(Page12 Lines 329-333, Page 15, Lines 409–412)

Reviewer #2

1, Because some patients contributed both eyes to the analysis, the observations are not statistically independent. Although the authors applied a generalized linear mixed model (GLMM) with patient ID as a random effect, the initial variable screening was performed using standard multivariable logistic regression at the eye level. It would be more appropriate to use the GLMM as the primary analysis from the outset.
 In addition, because the number of contamination events is very small in this study, multivariable logistic regression may be prone to overfitting and may not be statistically appropriate. The authors may consider limiting the analysis to univariable logistic regression and acknowledging in the limitations that multivariable analysis could not be reliably performed due to the limited number of events. The manuscript would also benefit from clearer reporting of how many patients underwent bilateral surgery and whether both eyes were included in the analysis.

Response:

Thank you for these important comments. We agree that the non-independence of observations should be appropriately addressed and that the small number of events limits the robustness of multivariable analyses.

First, we have revised the statistical approach to use a generalized linear mixed model (GLMM) with patient ID included as a random effect as the primary analytical method, thereby appropriately accounting for within-patient correlation. The previous eye-level multivariable logistic regression used for variable screening has been removed. (Page5 Lines175-180)

Second, considering the limited number of contamination events (n = 7), we have modified the analysis to focus primarily on univariable GLMM analyses. The results are now presented in a newly added Table 3.

Third, we have revised the Results section to clearly state that 96 eyes from 86 patients were included in the analysis, and that 10 patients underwent bilateral surgery with both eyes included. (page6 Line184-185)

Finally, we have strengthened the Limitations section to explicitly acknowledge that the small number of events may affect the stability of the model and that the results should be interpreted with caution.

Page14 Lines 387-391

2, The conclusion that contamination is “not completely avoidable” during 25-gauge pars plana vitrectomy may be somewhat overstated. Because this study was conducted at a single institution with specific surgical techniques and perioperative protocols, the findings may not necessarily be generalizable to all surgical settings.
Differences in surgical technique, operating room environment, infection control protocols, and surgeon experience may influence contamination rates. Therefore, the authors may wish to moderate the tone of the conclusion and state that contamination may occur during 25-gauge PPV rather than suggesting that it is inherently unavoidable.

Response:

Thank you for this insightful comment. We agree that the original wording of the conclusion may have been overly strong. We have revised the statement to adopt a more cautious tone, replacing “not completely avoidable” with “may still occur,” to avoid overgeneralization.

Page14 Lines 404-407

In addition, we have added a statement acknowledging that this was a single-center study conducted under specific surgical techniques and perioperative protocols, and that the generalizability of the findings may be limited. The revised conclusion now reflects these considerations. (page14 Lines 380-382)

3, The manuscript mentions the use of Phisoderm solution in patients with iodine allergy. Please provide a more detailed description of this solution (e.g., its active components). It would also be helpful to clarify whether this agent contains chlorhexidine or another antiseptic. In addition, please report the number or proportion of patients in whom this solution was used.

Response:

Thank you for this comment. Upon re-examining the clinical records, we confirmed that no patients with iodine allergy were included in this study. Therefore, Phisoderm solution was not used in any cases. We have removed the related description from the Methods section to avoid confusion.

4, The authors state that tropicamide phenylephrine hydrochloride was administered twice daily for 3–4 weeks after surgery. Please clarify the rationale for this postoperative treatment. (L162-163)

Response

We thank the reviewer for this important comment. We have revised the Methods section to clarify the postoperative use and duration of tropicamide phenylephrine hydrochloride.

In our standard postoperative regimen, antimicrobial eye drops (0.5% moxifloxacin), 0.1% betamethasone sodium phosphate, and bromfenac were administered for 3 to 4 weeks. In contrast, tropicamide phenylephrine hydrochloride was administered only for 1 week postoperatively. The purpose of this medication is to induce mydriasis and reduce the risk of posterior synechiae formation.

These details have been corrected and clarified in the revised manuscript.

Page5 Lines167-170

5, The Methods section should include information regarding the surgeons who performed the procedures and their level of surgical experience. Surgeon experience may influence the risk of intraoperative contamination. In complex cases such as PVR, PDR, and RRD, which actually required longer operative times and advanced surgical skills, the risk of contamination during surgery may be higher.

Response:

We thank the reviewer for this important comment. We have added information in the Methods section describing the surgeons who performed the procedures, noting that surgeries were performed by multiple vitreoretinal surgeons with varying levels of experience, ranging from less than one year of experience to experienced specialists.

In addition, we evaluated the association between surgeon and contamination using a generalized linear mixed model with patient ID as a random effect and found no significant difference in contamination rates among surgeons.

Page4 Lines132-134, page8 Lines 224-225

6, It would be important to clarify how many ports were used during the vitrectomy procedures. Specifically, please indicate whether the surgeries were performed using a standard three-port system or whether a four-port system with chandelier illumination was used in some cases.

Response

We thank the reviewer for this insightful comment. We have revised the manuscript to clarify the number of ports used during vitrectomy procedures.

In our cohort, 19 eyes underwent standard three-port vitrectomy, whereas 77 eyes underwent four-port vitrectomy with chandelier illumination. We further evaluated the association between port number and contamination and found that contamination was more frequently observed in three-port procedures.

These details have now been incorporated into the Results section. In addition, we have included a discussion of the potential mechanisms underlying this finding, particularly focusing on differences in instrument manipulation between three-port and four-port techniques.

Page6 Lines 192-193, Page11 Lines 296-306

7, The discussion of COVID-19–related changes in endophthalmitis incidence in the Introduction may not be directly relevant to the main focus of this study. The authors may wish to reconsider whether this information is necessary in the Introduction.

Response:

Thank you for this comment. We agree that this information is not directly relevant to the main focus of our study. Therefore, we have removed the discussion of COVID-19–related changes in endophthalmitis incidence from the Introduction to improve the focus and clarity of the manuscript.

8, Based on the Methods section, povidone-iodine irrigation appears to have been applied before trocar insertion; therefore, bacterial entry into the eye at the time of port insertion seems relatively unlikely. It would therefore be important to clarify where bacterial growth was most frequently detected on the cannulas. For example, was bacterial growth mainly detected on the outer surface of the cannula extending toward the tip, or within the inner lumen of the cannula? If possible, the distribution of bacterial growth along the cannula should be described.

Response:

Thank you for this important comment. Cannulas were embedded directly into the culture medium, which did not allow precise differentiation between contamination of the outer surface and the inner lumen. However, we carefully reviewed the distribution of bacterial colony formation and found that colonies were observed in different patterns, including around the distal tip, the proximal hub, or both regions, with no predominant pattern.

We have added this information to the revised manuscript and expanded the Discussion to consider multiple potential routes of contamination.

Page4 Lines141-144, page8 Lines203-205, Page11 Lines 271-280

9, On the other hand, in complex cases such as PVR, longer surgical duration and repeated intraoperative manipulations may increase the possibility of contamination. In the present study, contamination occurred in cases with RRD, PDR, and PVR, in which the surgical duration also appears to have been relatively longer. For example, during surgery the port may become partially dislodged and require reinsertion, or the surgeon may need to push the cannula back into position. Such additional manipulations could potentially introduce contamination. Therefore, it would be helpful if the authors could clarify whether any ports became dislodged and required reinsertion during surgery, and report the number of such events if they occurred. This information would help readers better interpret the potential mechanisms of contamination observed in this study.

Response:

Thank you for this important suggestion. We reviewed the intraoperative videos of all cases in which contamination was detected. No instances of port dislodgement or reinsertion were observed in any of these cases.

We have added this information to the revised manuscript. In addition, we have expanded the Discussion to consider other possible mechanisms of contamination, such as contact of surgical instruments with the ocular surface, surgical drape, or pooled irrigation fluid during repeated intraoperative manipulations.

Page8 Lines217-218, Page11 Lines 282-290

10, In particular, for the cases in which contamination was detected on surgical instruments such as the vitrectomy probe or cannula, it would be valuable to review the intraoperative videos, if available, to investigate the potential cause of contamination in greater detail. If the cause cannot be clearly identified even after such review, the authors should consider discussing other possible mechanisms in the Discussion section.
For example, contamination might occur if an instrument such as the light probe comes into contact with pooled irrigation fluid around the operative field or on the surgical drape and is then reinserted into the eye. Exploring such potential mechanisms would help readers better understand how contamination may occur during surgery.

Response:

Thank you for this insightful suggestion. We agree that reviewing intraoperative videos could provide valuable information regarding the mechanisms of contamination. However, intraoperative video recordings were not systematically reviewed for this purpose in the present study, and therefore we were unable to identify specific contamination events.

We have revised the Discussion to include a more detailed consideration of potential mechanisms of contamination during surgery, including contact of surgical instruments with pooled irrigation fluid or the surgical drape, as well as repeated instrument insertion and removal through the cannula.

Page11 Lines 282-290

11, It would also be clinically useful to evaluate whether contamination was associated with the number of instrument insertions through the port during surgery.

Response:

Thank you for this valuable suggestion. We agree that the number of instrument insertions through the port may be an important factor influencing the risk of contamination. However, this variable was not systematically recorded in the present study, and therefore we were unable to evaluate this association.

We have added a statement in the Discussion to acknowledge this limitation and to highlight the need for future studies to investigate the relationship between the frequency of instrument manipulation and contamination risk.

Page11 Lines 291-294

12, Please clarify the rationale for the procedure described in lines 138–141. If a light guide or similar device is inserted into the cannula, there is a possibility that bacteria could adhere to it. Even at this stage, bacteria attached to the endoilluminators, beveled probes, or trocars could potentially come into contact with each other, leading to cross-contamination. The authors should discuss whether this possibility may have affected the culture results.

Response:

Thank you for this important comment. The removal of cannulas together with the endoilluminators or beveled probes was part of our routine surgical practice for wound closure and was not specifically performed for the purpose of this study. We have clarified this point in the Methods section.

We agree that this procedure may introduce a potential risk of cross-contamination, as bacteria attached to one instrument could come into contact with another during removal. We have added a limitation addressing this possibility and its potential impact on the culture results.

Page4 Lines138-139, Page14 Line387-396

13, Previous studies by Shimada H (The author of Reference 7). and colleagues have many times reported that repeated povidone-iodine irrigation can reduce the risk of bacterial entry into the vitreous cavity. Please clarify whether such repeated iodine irrigation was performed in the present study.

Response:

Thank you for this important comment. In the present study, iodine irrigation was performed at specific time points, including immediately before sclerotomy creation and just before wound closure. In cases involving concomitant cataract surgery, the solution was also applied before intraocular lens implantation.

However, repeated intraoperative iodine irrigation, as described in previous studies, was not performed. We have clarified this point in the Methods section. (Page 4, Line 130)

14, More detailed information about the contaminated cases would be helpful. For example, among the cases of RRD, PDR, PVR, and lens dislocation, please specify which surgical instruments were contaminated. Presenting these data in a table would improve clarity. In addition, it would be useful to evaluate whether contamination was associated with factors such as laterality, surgeon handedness, or surgical quadrant. The manuscript also does not specify how many of these cases were emergency surgeries, which should be clarified.

Response:

We thank the reviewer for this valuable suggestion. We have added a new table (Table4) summarizing the detailed characteristics of the contaminated cases, including the indication for surgery, contaminated instruments, surgeon handedness, and whether the procedure was performed as an emergency surgery.

Regarding the surgical quadrant, the positional information of each instrument or cannula was not specifically recorded in this study, and therefore we were unable to determine which quadrant was associated with contamination. This limitation has been acknowledged in the revised manuscript. Future studies with detailed intraoperative recording may help clarify whether the spatial location of instrument use influences the risk of contamination.

Page8 Lines 217-221, Page14 Lines 396-398

15, The discussion in lines 274–291 suggests that routine culturing of surgical instruments may be beneficial. However, considering the very low incidence of postoperative endophthalmitis and the associated costs, routine culturing for all cases may not be practical in clinical settings. Based on the findings of this study, it may be more appropriate to focus the discussion on identifying higher-risk situations, such as cases in which preoperative antibiotic eye drops were not administered, rather than emphasizing the general usefulness of routine instrument cultures.

Response:

Thank you for this insightful comment. We agree that routine culturing of surgical instruments in all cases may not be practical given the low incidence of postoperative endophthalmitis and associated costs.

Accordingly, we have revised the Discussion to moderate our original statement and to emphasize the importance of identifying higher-risk situations, such as cases without preoperative antibiotic eye drops, rather than suggesting the general usefulness of routine instrument cultures.

Page13 Lines 360-363

16, Table 1 appears to contain an error in the age values of the non-contaminated group. The reported mean age and standard deviation (“19.43 (3.57)”) seem inconsistent with the overall cohort and likely represent a typographical error.

Response:

Thank you for carefully reviewing our manuscript and for pointing out this error. We recalculated the age values based on the original dataset, and the corrected values have been updated in Table 1. The correct mean age and standard deviation have now been revised in Table 1.

We have carefully rechecked all data presented in the tables to ensure accuracy.

17, Minor language and typographical issues should be corrected. For example, “obsereved” in the figure legend should be corrected to “observed”.

Response:

Thank you for your comment. We have corrected the typographical error (“obsereved” to “observed”) in the figure legend.

In addition, we have carefully reviewed the entire manuscript and corrected minor language and typographical errors to improve clarity and readability.

18, It may also be informative to include patients receiving immunosuppressive agents or chemotherapy in future studies to evaluate whether these conditions influence the risk of surgical instrument contamination. (L109-110)

Response:

Thank you for this valuable suggestion. We agree that patient-related factors such as immunosuppressive therapy or chemotherapy may influence the risk of surgical instrument contamination. We have added a statement in the Discussion to highlight the importance of evaluating these factors in future studies.

Page13 Lines 369-372

Reviewer #3

Issue 1. Interpretation of contamination versus clinical infection
A. Clinical relevance
Although bacterial contamination was detected in 7.3% of cases, no postoperative endophthalmitis occurred during the follow-up period. Therefore, the clinical significance of instrument contamination remains unclear.
The manuscript introduces postoperative endophthalmitis as a major concern in the Background section; however, the relationship between the detected contamination and the risk of endophthalmitis is insufficiently discussed.
In particular, the statement in the Conclusion that“The considerable rise in fluoroquinolone-resistant bacterial strains suggests that ophthalmologists should reassess their selection of preoperative antibacterial agents” appears to go well beyond what can be supported by the data presented in this study.

B. Relationship with bacterial load
Experimental studies using rabbit endophthalmitis models have suggested that intraocular infection typically requires a minimum infectious inoculum, often approximately 10²–10³ CFU depending on the bacterial species.
The mere detection of bacteria does not necessarily indicate a clinically meaningful infection risk. It would be informative to evaluate the bacterial load associated with the detected contamination, as well as its relationship with preoperative or intraoperative factors. Thus, quantification of bacterial burden (e.g., CFU counts) would help clarify whether the detected contamination could plausibly lead to postoperative endophthalmitis.

Response:

Thank you for these important comments. We agree that the detection of bacterial contamination does not necessarily indicate clinically meaningful infection, particularly in the absence of postoperative endophthalmitis in our cohort.

Accordingly, we have revised the Discussion to clarify that the clinical implications of contamination should be interpreted with caution and that infection typically requires a minimum infectious inoculum, as suggested by experimental studies.

We also acknowledge that bacterial load was not quantified in this study, which limits the ability to assess the relationship between contamination and infection risk. We have added this as a limitation and highlighted the importance of quantitative evaluation (e.g., CFU counts) in future studies.

In addition, we have revised the Conclusion to avoid overinterpretation of the findings regarding fluoroquinolone-resistant bacteria.

Page 10 Lines 247-262, Page15 Lines 409-412

Issue 2. Sample size and statistical power
Although the sample size of 96 eyes is adequate for estimating the contamination rate, the number of contamination events (7 cases) is very small for robust multivariable analysis. With such a limited number of events, the multivariable logistic regression and GLMM models may be unstable. The events-per-variable assumption (EPV < 10) is likely not satisfied, raising concerns about model overfitting. This concern is reflected in the wide confidence interval reported for the association between preoperative antibacterial eye drops and contamination (OR 0.13, 95% CI: 0.025–0.695).
Given the small number of contamination events, the robustness of the statistical analysis may be limited. The authors should explicitly acknowledge this limitation in the manuscript and interpret the results with caution. Alternatively, increasing the sample size or number of events in future studies would help strengthen the reliability of the findings.

Response:

Thank you for this important comment. We agree that the small number of contamination events may limit the robustness of the statistical analyses. In particular, the number of events per variable was low, which may increase the risk of model overfitting and contribute to the wide confidence intervals observed.

We have revised the Limitations section to explicitly acknowledge these issues and to emphasize that the results should be interpreted with caution. We also agree that studies with larger sample sizes and a greater number of events are needed to confirm our findings.

Page14 Lines 387-391

We sincerely thank the Academic Editor and the reviewers for their insightful and constructive comments. We believe that the revisions made in response to these comments have significantly improved the clarity, rigor, and overall quality of our manuscript. We hope that the revised version meets the requirements for publication in PLOS ONE, and we respectfully submit it for your consideration.
