# Supplementary material for: Bacterial contamination of surgical instruments in 25-gauge pars plana vitrectomy: Incidence and risk factors
Source: PLoS One. 2026 May 28;21(5):e0349663. doi: 10.1371/journal.pone.0349663 (PMC13218455; doi:10.1371/journal.pone.0349663)
Supplement: Attachment — Submitted filename: Response to Reviewers .docx [file pone.0349663.s003.docx]

Dear Editor and Reviewers,

We sincerely thank the Academic Editor and the reviewers for their thorough evaluation of our manuscript and for their valuable and constructive comments. We greatly appreciate the time and effort devoted to reviewing our work.

We have carefully considered all comments and have revised the manuscript accordingly. We believe that these revisions have improved the clarity, robustness, and overall quality of the study. In the following sections, we provide a detailed, point-by-point response to each comment. In the revised manuscript with track changes, deletions are shown using strikethrough, and additions are indicated in red.

Journal Requirements:

1. If the reviewer comments include a recommendation to cite specific previously published works, please review and evaluate these publications to determine whether they are relevant and should be cited. There is no requirement to cite these works unless the editor has indicated otherwise. 

2. Please review your reference list to ensure that it is complete and correct. If you have cited papers that have been retracted, please include the rationale for doing so in the manuscript text, or remove these references and replace them with relevant current references. Any changes to the reference list should be mentioned in the rebuttal letter that accompanies your revised manuscript. If you need to cite a retracted article, indicate the article’s retracted status in the References list and also include a citation and full reference for the retraction notice.

Response:

Thank you for this instruction. Upon careful review of the reference list, we identified that an inappropriate reference had been cited. We have therefore replaced Reference 16 with a more relevant study (Shockley et al., 1985), which directly supports the statement regarding the minimum infectious inoculum required for the development of endophthalmitis. The reference list has been revised accordingly.

Review Comments to the Author

Reviewer #1: The authors have responded appropriately and thoroughly to the reviewer’s comments, and the manuscript is very well written overall. Each point raised has been carefully addressed with clear and sufficient explanations, demonstrating the authors’ strong understanding of the subject and their commitment to improving the quality of the work. The revisions have significantly enhanced the clarity, consistency, and scientific rigor of the manuscript. In particular, the additional details and clarifications provided in response to the comments have improved the transparency and reproducibility of the study. The structure is logical, and the arguments are presented in a coherent and persuasive manner. Overall, the manuscript is now in excellent condition and suitable for publication.

Response:

We sincerely thank the reviewer for the positive and encouraging comments. We greatly appreciate the reviewer’s careful evaluation of our manuscript and are pleased that the revisions have improved the clarity, consistency, and scientific rigor of the study. We are grateful for the reviewer’s recognition of our efforts to enhance the quality of the manuscript.

Reviewer #2: I appreciate the opportunity to review this revised manuscript. The authors have addressed many of the previous comments, particularly regarding the statistical analysis and clarification of surgical procedures, which has improved the manuscript.
However, some issues remain insufficiently addressed, including surgeon-related factors and the interpretation of results, which appears somewhat overstated, particularly in the Abstract. Addressing these points would be needed.

1, The role of surgeon-related factors remains insufficiently addressed. Please include the surgeons’ years of experience in Table 4.

Response:

We thank the reviewer for this important comment. In response, we have added information on surgeons’ years of experience to Table 4 to provide a more detailed characterization of the contaminated cases.

2, In addition, the manuscript does not clearly specify how many surgeons were involved in this study. This information should be explicitly reported.

Response:

We thank the reviewer for this important comment. We have revised the Methods section to clearly specify the number of surgeons involved in the study. A total of five vitreoretinal surgeons performed the procedures. This information has been added to the revised manuscript.

Page 4 Line 132

3, It would also be valuable to evaluate the association between surgeon experience and contamination in Tables 1 and 3. If analysis as a continuous variable is difficult, categorizing surgeons into groups (e.g., experienced vs. less experienced, such as ≥4–5 years) may be more appropriate. Furthermore, the variable “Surgeons” in Table 3 is unclear and should be clarified.

Response:

We thank the reviewer for this important and helpful suggestion. In accordance with the comment, we evaluated the association between surgeon experience and contamination. Surgeons were categorized into two groups based on their experience in vitreoretinal surgery (≥5 years and <5 years), as suggested. This variable has been incorporated into Tables 1 and 3, and the corresponding analysis has been added to the Results section. No significant association was observed between surgeon experience and contamination.

In addition, the variable previously labeled as “Surgeons” in Table 3 has been clarified and revised to “Surgeon experience (≥5 vs <5 years)” to improve clarity and consistency throughout the manuscript.

Page4 Lines 133-136, Page6 Lines 202-204, Page8 Lines 228-229

4, Throughout the manuscript, there are too many short paragraphs. Consolidating them into more meaningful and structured paragraphs would improve readability, particularly in the Discussion section.

Response:

We thank the reviewer for this helpful comment. We have revised the Discussion section by consolidating shorter paragraphs into more structured and cohesive paragraphs to improve readability and the overall flow of the text. These changes have enhanced the clarity and logical organization of the manuscript.

5, The conclusion that contamination is “not completely avoidable” appears not to have been revised in the Abstract. This statement should be moderated to better reflect the limitations of the study.

Response:

We thank the reviewer for this important comment. We have revised the Conclusion in the Abstract to moderate the tone and better reflect the limitations of the study. Specifically, the statement “not completely avoidable” has been replaced with “may occur.”

Page 2 Line50

Reviewer #3: Manuscript has been improved with the additional remarks on strengths and limitations of this study. I have no further comment.

Response:

We sincerely thank the reviewer for the positive and encouraging comments. We are pleased that the revisions, including the additional discussion on the strengths and limitations of the study, have improved the manuscript. We greatly appreciate the reviewer’s evaluation.

We sincerely thank the Academic Editor and the reviewers for their insightful and constructive comments. We believe that the revisions made in response to these comments have significantly improved the clarity, rigor, and overall quality of our manuscript. We hope that the revised version meets the requirements for publication in PLOS ONE, and we respectfully submit it for your consideration.
